# Supplementary material for: Tetrapeptide Ac-HAEE-NH2 Protects α4β2 nAChR from Inhibition by Aβ
Source: Int J Mol Sci. 2020 Aug 29;21(17):6272. doi: 10.3390/ijms21176272 (PMC7504039; doi:10.3390/ijms21176272)
Supplement: Supplementary file 1 [file ijms-21-06272-s001.zip › Supplementary Material.pdf]

Supplementary Figures

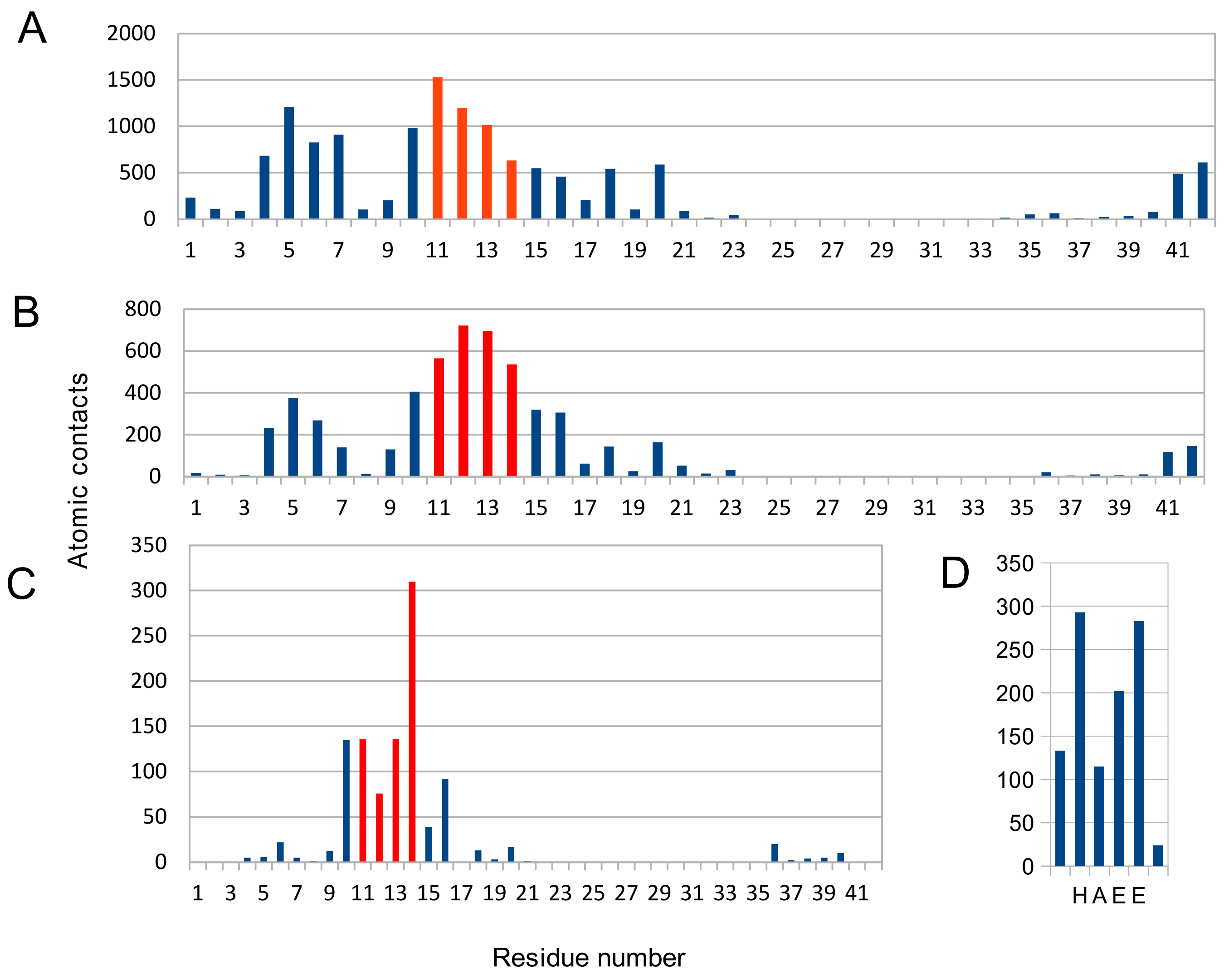

Figure S1. Docking results of Ac-HAEE-NH_2 to $A\beta_{42}$. Horizontally represented are amino acid residues 1-42 of $A\beta_{42}$, vertically - the total number of atomic contacts of Ac-HAEE-NH_2 in all models with each residue. Bars above the ^{11}EVHH^{14} site are highlighted in red. A - Fullblind docking, B - Targeted global docking; C, D - Binding information for 8 selected docking structures of Ac-HAEE-NH_2 to $A\beta_{42}$.

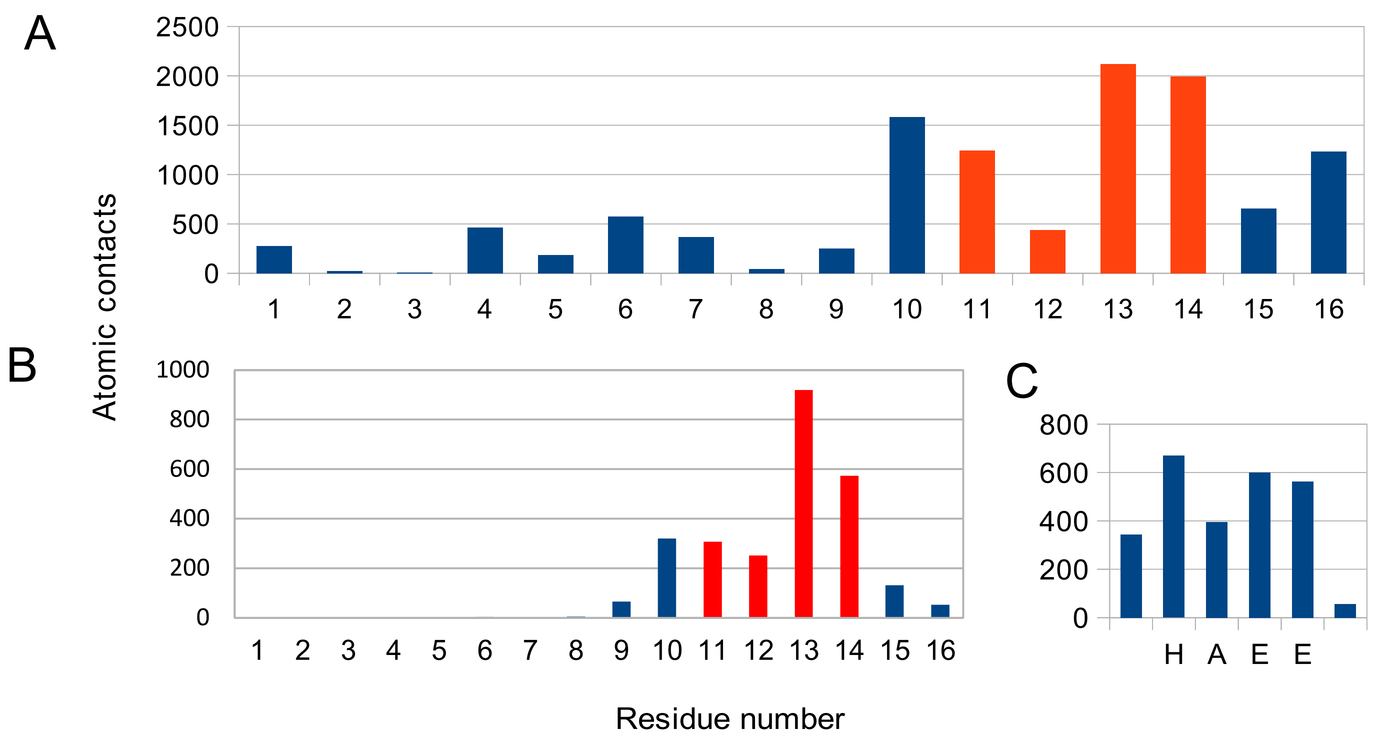

Figure S2. Docking results of Ac-HAEE-NH_2 to $A\beta_{16}$. Horizontally represented are amino acid residues 1-16 of $A\beta_{16}$, vertically - the total number of atomic contacts of Ac-HAEE-NH_2 in all models with each residue. Bars above the ^{11}EVHH^{14} site are highlighted in red. A - Targeted docking of Ac-HAEE-NH_2 to $A\beta_{16}$, B, C - Binding information for 22 selected structures of Ac-HAEE-NH_2 docking to $A\beta_{16}$.

Supplementary Tables

Table S1. The peptides with predicted charge complementarity (both in a parallel and anti-parallel orientation) for ^{11}EVHH^{14} region of Aβ tested in the direct binding assay. The peptides with the strongest binding to $Aβ_{16}$ by Kd are highlighted in yellow

Number	Sequence	Number	Sequence
#1	Ac-HADD-NH_2	#13	Ac-HADE-NH_2
#2	Ac-DDAH-NH_2	#14	Ac-EDAH-NH_2
#3	Ac-KADD-NH_2	#15	Ac-KADE-NH_2
#4	Ac-DDAK-NH_2	#16	Ac-EDAK-NH_2
#5	Ac-RADD-NH_2	#17	Ac-RADE-NH_2
#6	Ac-DDAR-NH_2	#18	Ac-EDAR-NH_2
#7	Ac-HAED-NH_2	#19	Ac-HAEE-NH_2
#8	Ac-DEAH-NH_2	#20	Ac-EEAH-NH_2
#9	Ac-KAED-NH_2	#21	Ac-KAEE-NH_2
#10	Ac-DEAK-NH_2	#22	Ac-EEAK-NH_2
#11	Ac-RAED-NH_2	#23	Ac-RAEE-NH_2
#12	Ac-DEAR-NH_2	#24	Ac-EEAR-NH_2

Tables S2. Kinetic parameters for interaction of immobilized $A\beta_{16}$ with different charge-complementary peptides at 25 °C in 10 mM HEPES, pH 6.8. The calcaluation of kinetic parameters for all the analytes was impossible due to weak binding for some peptides. The most potent binders are highlighted in yellow. Data are presented as a mean±SD of three independent measurements.

Peptide sample	$K_d \times 10^4$ M	$K_a \times 10^{-2}$ M^{-1}	$k_{on} \times M^{-1}s^{-1}$	$k_{off} \times 10^{-3}$ s^{-1}
#1	32.5±0.9	3.08±0.08	3.20±0.08	10.40±0.02
#2	297±8	3.37±0.09	0.52±0.01	15.4±0.1
#4	13.7±0.5	7.3±0.3	4.57±0.09	6.2±0.1
Ac-RADD-NH_2 #5	0.13±0.02	800±100	18.5±0.1 e1	0.24±0.04
#9	33±7	3.1±0.6	5.7±0.9	19±1
#12	19.8±0.7	5.1±0.2	3.58±0.08	7.09±0.09
#15	45±3	2.2±0.1	2.2±0.1	9.6±0.2
#16	710±50	0.141±0.009	0.136±0.006	9.7±0.2
#18	350±30	0.28±0.03	0.13±0.01	4.7±0.1
Ac-HAEE-NH_2 #19	0.9±0.3	110±40	0.37±0.05	0.035±0.008
Ac-RAEE-NH_2 #23	77±6	1.3±0.1	1.5±0.1	11.20±0.08
